# Possibility of Autonomous Estimation of Shiba Goat’s Estrus and Non-Estrus Behavior by Machine Learning Methods

**DOI:** 10.3390/ani10050771

**Published:** 2020-04-29

**Authors:** Toshiya Arakawa

**Affiliations:** Department of Mechanical Systems Engineering, Aichi University of Technology, Gamagori-shi, Aichi 443-0047, Japan; arakawa-toshiya@aut.ac.jp; Tel.: +81-533-68-1135

**Keywords:** shiba goat, Estrus, estimation, machine learning

## Abstract

**Simple Summary:**

Direct observation of mammalian behavior requires a substantial amount of effort and time, particularly if the number of animals to be observed is sufficiently large or if the observation is conducted for a prolonged period. In this study, different machine learning methods were applied to detect and estimate whether a goat is in estrus, based on the goat’s behavior. The percentage concordance (PC) of their behavior, based on tracking data and human observations, was evaluated. The results establish that HMM is an adequate method from the viewpoints of estimation, statistical, and time series modeling. In this experiment, neural network did not seem to be adequate method, however, if the more goat’s data were acquired, neural network would be an adequate method for estimation.

**Abstract:**

Mammalian behavior is typically monitored by observation. However, direct observation requires a substantial amount of effort and time, if the number of mammals to be observed is sufficiently large or if the observation is conducted for a prolonged period. In this study, machine learning methods as hidden Markov models (HMMs), random forests, support vector machines (SVMs), and neural networks, were applied to detect and estimate whether a goat is in estrus based on the goat’s behavior; thus, the adequacy of the method was verified. Goat’s tracking data was obtained using a video tracking system and used to estimate whether they, which are in “estrus” or “non-estrus”, were in either states: “approaching the male”, or “standing near the male”. Totally, the PC of random forest seems to be the highest. However, The percentage concordance (PC) value besides the goats whose data were used for training data sets is relatively low. It is suggested that random forest tend to over-fit to training data. Besides random forest, the PC of HMMs and SVMs is high. However, considering the calculation time and HMM’s advantage in that it is a time series model, HMM is better method. The PC of neural network is totally low, however, if the more goat’s data were acquired, neural network would be an adequate method for estimation.

## 1. Introduction

The purpose of this study was to show that machine learning can be applied to detect and estimate whether a goat is in estrus based on the goat’s behavior, and the adequacy of detection and estimation method by machine learning is verified. Estrus is a state of sexual receptivity during which the female will accept the male and is capable of conceiving. This behavioral state occurs under hormonal regulation involving the ovary and pituitary gland, and precedes or coincides with ovulation [1]. Behaviors exhibited during estrus are significant for successful mating in all mammals. Estrus behavior is characterized by three components: attractivity, receptivity, and proceptivity [2]. Attractivity is the female’s stimulus value in evoking sexual responses by the male [2]; an example can be changes in the female’s scent (pheromones) [3]. Receptivity is defined as female responses that are necessary and sufficient for the male’s success in achieving intravaginal ejaculation [2]. Proceptivity is any behavior exhibited by a female that initiates or maintains sexual interaction with a male, which in goats includes approaching males, sniffing, mounting, and tail wagging [4,5,6]. Here, upon sniffing and mounting, the female permits mounting by the male. These behaviors are generally monitored by a human observer because direct observation is currently regarded as the best method for obtaining detailed data regarding specific behaviors [7]. Generally, direct observation requires substantial effort and time, if the number of targets to be observed is large and the observations are to be conducted for a prolonged period. For example, a previous study shows that it takes 24 h/day for 13 days to describe breeding behavior and activity budgets of an undisturbed pair of adult polar bears [8]. The case of goats is almost similar to this. In addition, observation-based data are based on a human observer’s subjective judgment, and the observation by other observers might yield different results. In other words, the observation results differ depending on the observers, particularly their skill or experience. For this reason, it is necessary to develop objective and effective quantitative evaluation methods to replace ineffective evaluation methods based on an observer’s direct observation. In the livestock industry in particular, the need for information and communication technology (ICT) and the Internet of Things (IoT) is a system of interrelated computing devices, mechanical and digital machines, objects, animals, or people that are provided with unique identifiers and the ability to transfer data over a network without requiring human-to-human or human-to-computer interaction [9]) for monitoring and management of production, mating, and health of animals has been growing [10].

Previous studies [7] have shown that automated video tracking systems for studying animal behavior were introduced in the early 1990s and that they have been increasingly incorporated into studying laboratory mice [11,12,13] and other small animals. In addition to the development of digital image processing technology, several types of specialized computer software for animal tracking have been proposed, which allow tracking of positions and movement of subjects automatically and generate coordinates of a target position as time series data. Vector analysis of the trajectory data, i.e., the subject’s position, can be used to assess the activity of the subject by calculating movement distance and speed. More complex behaviors, such as social interactions between male and female mice can be identified automatically using statistical models [14,15,16]. The merit of quantitative evaluation methods is that they are not limited by an observer’s capacity and proficiency. Even if well-trained and proficient observers judge the behavior, the judgment might vary between two observers. In addition, even if the observers are well-trained, they might make mistakes, i.e. human error. According to our previous study, the machine learning method could detect states of mice that well-trained observers could not judge well due to human error [17]. We think that the case of estimating goat states is similar. Considering this, quantitative evaluation method has merit in terms of solving these problems.

The video tracking systems are widely used to track small objects, such as laboratory mice [12,14,15,16], small animals [18,19] and fishes [20], to large animals, such as monkeys [21] and pigs [22].

On the other hand, from the point of effective behavior analysis of large animals such as goats, tracking the animal’s behavior and analyzing its tracking data can be beneficial. For example, previous studies have involved fixing a data logger to cattle and detecting their estrus behavior, in cases of large animals’ behavior detection, and that data logger has been commercialized [23,24]. In addition, statistical modeling can be quite effective towards utilizing the recorded animal’s behavioral data. Previous studies show effective behavioral analysis and tag development from the point of statistical modeling [25]. However, it takes time and effort to attach sensors because the sensors are affixed to the animals by winding a band around them. In addition, there is a need to improve the materials used in sensors to prevent breakage by animals and shifting of attached sensors. It is also necessary to downsize sensors and facilitate sensor attachment considering field use [10], because it becomes difficult for animals to move around if the sensors are large in size, and it takes a long time, if attaching the sensor is not facilitated. This prevents efficient data acquisition.

Therefore, alternative methods for identifying estrus behavior in large animals must be developed. Video tracking is one of these methods because it does not restrict the animals’ movement due to attached sensors. Tracking data are acquired by attaching a marker to a goat (which is considered as an example of a large animal) and methods for estimating the goat’s estrus behavior from these data are devised. A previous study has shown that an estrus goat has an increased tendency to approach and stand near a male goat’s paddock, based on the male goat’s behavior [7]. In this case, the female goat’s behavior is recorded as a movie, and a professional evaluator judges the goat’s behavior subjectively for exhibiting estrus tendencies in each frame.

Generally, machine learning is used for pattern recognition [26] or estimation [27]. One application of machine learning is to estimate behavior. For example, machine learning is used for prediction of ambulation behavior [28] or everyday life activities [29]. As for animals’ behavior, it is used for automated measurement of social behavior of mice with depth sensing and video tracking [30]. In another study, dairy cows’ behavior is predicted by variable segmentation and ensemble classifiers [31]. It is suggested that machine learning could be applied to detect and estimate, and/or predict a goat’s behavior as well. Especially, as far as author’s survey, there are no previous studies about machine learning methods that estimate whether goat is in estrus state or not. In this study, as methods of machine learning, hidden Markov models (HMMs) [32,33,34], random forest, support vector machines (SVMs), and neural networks was applied to detect and estimate whether a goat is in estrus, based on the goat’s behavior, and the adequacy of this detection and estimation method is verified. As for HMMs, a previous study has shown that HMMs are adequate for detecting and estimating a large animal’s “estrus” and “non-estrus” behaviors [10]. However, in this study, “estrus” behavior is divided into “approaching the male” state and “standing near the male” state (this will be described in Section 2.4). The purpose of dividing goat’s behavior into two is for estimating more detail states of goats. Here, there are other machine learning methods asides from HMMs, and if we can estimate when these states related to “estrus” behavior occur by some machine learning method, it might help breeding management to mate animals successfully. This paper can contribute to this. In general, for breeding goats, it is said that different areas are needed if a large number of animals should be kept, and they should be divided into groups. Ideally, they are divided into groups, such as seeded male goats, young male goats, young female goats, mother and child goats (calving to weaning), adult female goats (unconceived), and sick goats. At mating time, sired goats are placed into the female flock [35].

It is important to detect estrus accurately because the breeding season will be over if estrus is missed. For this reason, breeders need to determine if their goats are in estrus. They need to train to discern whether goats are in estrus or not accurately. By demonstrating the possibility of using machine learning methods, such as those in this manuscript, to estimate goat behavior, breeders who are uncertain about their goats’ states will be able to learn about goat behavior in real time, eliminating the need to spend their time training to distinguish between estrus and non-estrus accurately.

The remainder of this paper is organized as follows. Section 2 comprehensively explains the experiments and the estimation methods used in this study, Section 3 describes the estimation results and discusses the results, and Section 4 summarizes the main findings of this research with a brief consideration for future study.

## 2. Materials and Methods

The methods described in the study by Endo et al. [7] are followed in this study.

### 2.1. Ethics Approval and Consent to Participate

Adult female Shiba goats used in this study were maintained at the Tokyo University of Agriculture and Technology, and all of the experimental procedures were approved by the University Committee for the Use and Care of Animals at Tokyo University of Agriculture and Technology (#27-18).

### 2.2. Animals and Housing

Six to eight goats were housed in a paddock with an outside area of 25 m^2^, a sheltered area of 15 m^2^, and a natural photoperiod. The goats were fed maintenance diets of alfalfa hay cubes twice a day (0900 h and 1500 h). Clean water and mineralized salt were provided *ad libitum*.

### 2.3. Video Recording

**Figure 1:**
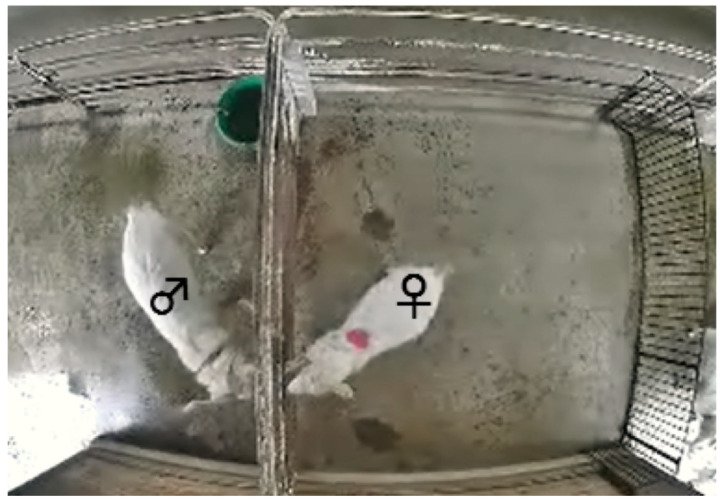
Image from the video recording data. One side of the observation pen was adjacent to the male paddock. A marker was attached to the back of the female goat to track its movements.

The study was conducted between November 2014 and January 2015. All female goats were checked for estrus once or twice daily and were considered to be in estrus when they allowed mounting by a male goat. Here, female goats and a male goat are housed separately. Between December 2014 and January 2015, 16 goats were tested (age = 2–9 years, body weight = 21–37 kg) and confirmed to be in estrus (n = 8: #24, #23, #17, #33, #12, #9, #4, and #22) or not in estrus (n = 8: #25, #13, #6, #3, #14, #15, #21, and #35). An observation pen (2.5 m × 2.5 m) was set up at a corner of the female paddock, with one side adjacent to the male paddock. A network camera (DG-SF334, Panasonic Corporation, Kadoma, Japan) was fixed to the ceiling. A captured image from the video recording data is shown in Figure 1.

Before beginning the observations, one male goat was tied loosely with a rope to the adjacent side of the observation pen. A bright-colored circle marker (red or blue, 12 cm in diameter) was attached to the back of the female goats to enable identification and tracking in video recordings. The female goats were then moved gently into the observation pen by one observer. The female and male goats were allowed to contact each other partially by poking their muzzles through the bars on the adjacent side of the observation pen. After an adaptation period of approximately 10 min, the behaviors of the goats were recorded for 10 min using a network camera recorder (BB-HNP17, Panasonic) connected to a personal computer. The computer was located in a building at a distance from the goat paddocks. During video recordings, the observer remained in the building to prevent disturbing the behaviors of the goats. Here, each goat was observed and their behavior was recorded individually.

### 2.4. Behavioral Analysis by Human Observation

After finishing the video recording, behavioral observations were performed once by a well-trained observer, who is a veterinary expert, using the video recordings. On the basis of previous descriptions of sexual behaviors during estrus in goats [7], the occurrence of two behaviors, “approaching the male” and “staying near the male,” were investigated as indicators of proceptivity. The following criteria was used to define each behavior:

- Approaching to the male
  Walking or running from the other side of the observation pen toward the area adjacent to the male paddock.
- Standing near the male
  Standing or behaving restlessly (continuously) in the area adjacent to the male paddock, and occasionally contacting the male through the bars of the pen.

The data frames were scored as 0 (behavior besides approaching and standing), 1 (approaching the male), or 2 (standing near the male) by the observer to analyze each behavior in terms of its frequency or duration. The continuous time was taken as the duration of the behavior. The video recordings were processed using ABDigitizer software (Chinou Jouhou Shisutemu, Kyoto, Japan). Here, we used large, visible markers to track the goats’ movements; however, this was because we used the ABDigitizer. Needless to say, even small devices, such as RFID tags, could be used to track the goats’ movements. Any device that can track the goats’ movements could be used. As for judgment whether goat is in estrus or non-estrus, receptivity symptoms, such as a flush look to the vulva, outflowing of swelling and mucus, loss of appetite, increase in the frequency of urination, and characteristic cries, were used.

**Figure 2:**
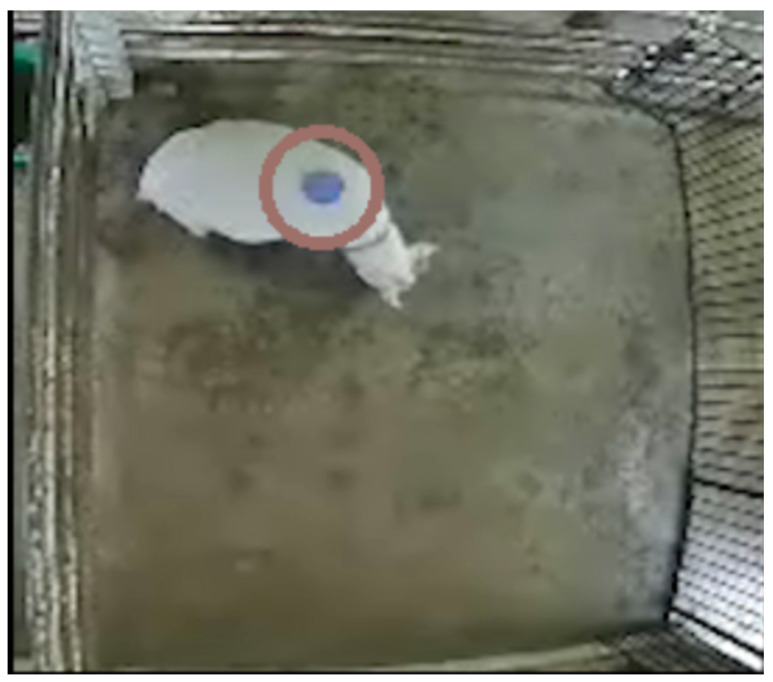
Image from the video recording data. One side of the observation pen was adjacent to the male paddock. A marker was attached to the back of the female goat to track goat movements.

The positions and movements of the markers attached to the back of the goats were tracked automatically (Figure 2), and the x- and y-coordinates of the central point of the marker were outputted every 0.5 s. Thus, a 10-min video tracking dataset contained 1200 frames.

In this context, it was considered that a goat pair assumed one of three states $s_{k}$ at each time point *k* (1, ..., 1200):

$s_{k}$ = 0: behavior besides approaching and standing (state 0),
$s_{k}$ = 1: approaching the male (state 1),
$s_{k}$ = 2: standing near the male (state 2)

where $s_{k}$ is the state of the pair at time point *k*. The reason for considering these three states was that the human observer identified three behavioral states, and the ability of the model to classify those three states correctly needed to be assessed.

Next, observable variables were calculated. The number of observable variables in this model were three:

**(a)**  x-coordinate: x(k)
**(b)**  y-coordinate: y(k)
**(c)**  step length of a goat at k and k+1: $L\left(k\right)=\sqrt{{\left\{x\left(k+1\right)-x\left(k\right)\right\}}^{2}+{\left\{y\left(k+1\right)-y\left(k\right)\right\}}^{2}}$

Here, the reason for adopting these three observable variables was based on a previous study [10] (see Appendix A).

### 2.5. Modeling and Estimation of Estrus Behavior by Hmm

An HMM is composed of a Markov process that updates “states” and a (conditional) probabilistic distribution of the observable variables in a given state. The principal purpose of applying an HMM was to estimate behavioral states from a time series of observable variables.

**Figure 3:**
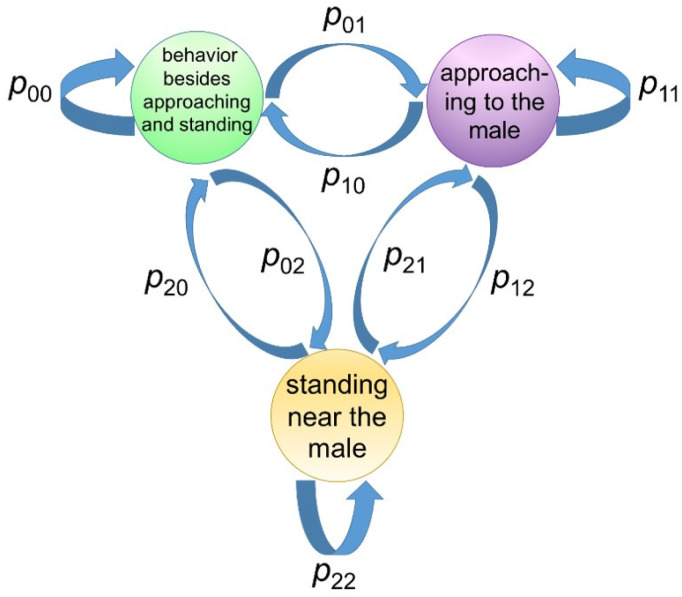
Three-state HMM. Shiba goats show three states, behavior besides approaching and standing, approaching the male, and standing near the male. There is a timing when the state remains the same, and there is a timing when the state transitions to another state.

The state was considered to be updated according to Markov process (Figure 3).

Next, the conditional distribution p(y|s) of observable variables for a given state was explained, where *y* was the set of observable variables and *s* is the state. For all the goats, the state *s* during each data frame was labeled by the observer, thus p(y|s) was easily calculated if *y* was observed.

**Figure 4:**
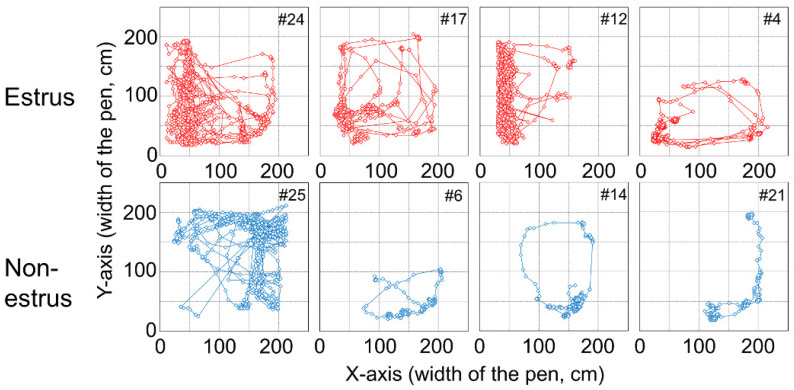
Tracking data of female goats in estrus and non-estrus. The *x*-coordinate of a female goat tends to be smaller when the female goat is in estrus and larger when the goat is in non-estrus.

In addition, Figure 4 shows the tracking data of the female goat’s movement from the experiment. Figure 4 shows tendencies for the *x*-coordinate of an estrus goat to be smaller, indicating that a female goat approaches the male goat in estrus. On the other hand, the *x*-coordinate of a non-estrus goat tends to be larger, indicating that a female goat which is not in estrus, approaches the opposite side of the male goat. Considering these results and the previous study [7], the position, i.e., *x*- and *y*-coordinates of female goats seemed to be adequate for observable variables. Step length of a goat, which could be considered as an observable variable from the result of the observation, was also included.

Thus, the observed $z_{k}$ at time point *k* is the three-dimensional vector $z_{k}=\left(x\left(k\right),y\left(k\right),L\left(k\right)\right)$. The number of sets of $z_{k}$ given was 1200 × 3, because each set of eight goats had 1200 data frames and three observable variables. These sets were divided into three groups, based on whether the state exhibited was any behavior besides approaching and standing (state = 0), approaching the male (state = 1), or standing near the male (state = 2), respectively.

It is assumed that $z_{k}$ adhered to the distribution $p(z_{k}|s_{k}=0)$ if the state was “behavior besides approaching and standing,” $p(z_{k}|s_{k}=1)$ if the state is “approaching the male,” and $p(z_{k}|s_{k}=2)$ if the state is “standing near the male.” $p(z_{k}|s_{k}=0)$, $p(z_{k}|s_{k}=1)$ and $p(z_{k}|s_{k}=2)$ were calculated based on three observable values and scores by the observer. Here, it was assumed that the observable variables conformed to Gaussian distribution, based on our previous studies [10,36].

The state $s_{k}$ was updated in accordance with a Markov model, as determined by the Markov transition matrix *T*:

$$T=\left[\begin{array}{ccc} p_{00} & p_{01} & p_{02}\\ p_{10} & p_{11} & p_{12}\\ p_{20} & p_{21} & p_{22} \end{array}\right]$$

The Markov transition matrix *T* was estimated by counting the transitions between the states 0→0,0→1,0→2,1→0,1→1,1→2,2→0,2→1, and 2→2 for all eight pairs. This was the model that was used to determine the behavioral state of the goats, in place of a human observer. To implement this model, *T*, $p(z_{k}|s_{k}=0)$, $p(z_{k}|s_{k}=1)$, and $p(z_{k}|s_{k}=2)$ were assumed. Based on the training data (eight pairs formed out of 16 goats, of which four were in estrus and the others were in non-estrus), the Markov transition matrix *T* and the conditional distribution of observable variables $p(z_{k}|s_{k}=0)$, $p(z_{k}|s_{k}=1)$, and $p(z_{k}|s_{k}=2)$ using a Gaussian mixture model were estimated, where the variance–covariance matrix of each component Gaussian was assumed to be diagonal. Data from the estrus goats, namely, #24, #23, #17, and #33, and non-estrus goats, namely, #25, #13, #3, and #35, were used to construct a Gaussian mixture model (i.e., they were used as training models). There were six transition probabilities, one mean, and one variance for each Gaussian component of the state-dependent observation distributions. The component number of the Gaussian mixture model was selected using Akaike’s Information Criterion (AIC) [10,36]. It was found that the number of Gaussian elements in states 0, 1, and 2 was 10 by AIC. On the other hand, the other data from estrus goats, namely, #12, #9, #4, and #22, and non-estrus goats, namely, #6, #14, #15, and #21, were used as test data. In other words, (1200×8)×(3+1) size matrix data, i.e., 38,400 data, was used for both the training and test data. It may be true that non-estrus data does not need to be included for the training group because the state which is not judged as estrus can be judged as non-estrus. However, if we include non-estrus data for the training group, the estimation accuracy is expected to improve because the behavior on non-estrus goats is also used for training. Non-estrus data was therefore included for the training group in this manuscript.

Next, using the tracking data from goats, $s_{1},\ldots ,s_{1200}$ were estimated as follows. The sequence, $z_{1},\ldots ,z_{k}$ of observable variables, was denoted up to time point *k* by $Z_{k}$. First, the entire sequence $Z_{1200}=\left(z_{1},\ldots ,z_{1200}\right)$ of observable variables, was computed from the tracking data. The application of standard formulas of the HMM allowed the computation of the posteriori distribution $p(s_{k}|Z_{1200})$ of the state as explained below, and the state $s_{k}$ at point *k* was determined as the model

$$s_{k}=\underset{s_{k}}{\arg\;\max}\;p\left(s_{k}|Z_{1200}\right)$$

of $p(s_{k}|Z_{1200})$.

The sequence $s_{1},\ldots ,s_{1200}$ of states, estimated using the HMM was used in place of the sequence of states that would have been obtained by a human experimenter, and the associated Markov probability was obtained.

To compute $p(s_{k}|Z_{1200})$, $p(s_{k}|Z_{k})$ was computed according to the following recursive formula:

(1) $$p\left(s_{k}|Z_{k-1}\right)=\sum_{s_{k-1}}p\left(s_{k}|s_{k-1}\right)p\left(s_{k-1}|Z_{k-1}\right)$$

(2) $$p\left(s_{k}|Z_{k}\right)=\frac{p\left(z_{k}|s_{k}\right)p\left(s_{k}|Z_{k-1}\right)}{\sum_{s_{k}}p\left(z_{k}|s_{k}\right)p\left(s_{k}|Z_{k-1}\right)}$$

Equations (1) and (2) were applied recursively to compute $p\left(s_{k-1}|Z_{k-1}\right)\to p\left(s_{k}|Z_{k-1}\right)\to p\left(s_{k}|Y_{k}\right)\to p\left(s_{k+1}|Y_{k}\right)\to ...$. Here, it was noted that $p(s_{k}|s_{k-1})$ comprised only of the elements in the Markov transition matrix *T*, and $p(z_{k}|s_{k})$ was estimated with the Gaussian mixture model. After $p(s_{k}|Z_{k})$ had been computed, $p(s_{k}|Z_{1200})$ was computed according to the following backward recursive formula:

(3) $$p\left(s_{k}|Z_{1200}\right)=p\left(s_{k}|Z_{k}\right)\sum_{s_{k+1}}\frac{p\left(s_{k+1}|Z_{1200}\right)p\left(s_{k+1}|s_{k}\right)}{p(s_{k+1}|Z_{k})}$$

Refer, Kitagawa [37] for the derivation of (1), (2), and (3), for instance. These equations were calculated using MATLAB 2014a, and the HMM algorithms were implemented by the author. Hence, packages pertaining to HMM were not used.

### 2.6. Modeling and Estimation of Estrus Behavior by Random Forest

For comparison of estimation accuracy, machine learning methods apart from HMM were considered. The difference HMM and machine learning methods apart from HMM is that the latter does not consider time transition and time-series. However, both the former and the latter is as the same at the point of considering that these handle (1200×16)×(3+1) size matrix data. Here, 1200 means the frame number of each goats, 16 means total number of goats, 3 means observable variables, and 1 means the result of detection through human observation. Thus, the following machine learning methods handle these matrix data. Here, as for modeling by machine learning, the data from estrus goats, namely, #24, #23, #17, and #33, and non-estrus goats, namely, #25, #13, #3, and #35, were used as training data, similar to the construction of a Gaussian mixture model.

Random forest was a data construct applied to machine learning that developed a considerable number of random decision trees, which analyzed sets of variables. This type of algorithm helped enhance the ways in which technology analyzed complex data [38]. Random forest was applied to classification [39], detection [40] and so on.

Algorithm of random forest is consisted of the following four steps [41]:

(1) First, start with the selection of random samples from a given dataset.
(2) Next, this algorithm will construct a decision tree for every sample. Then it will get the prediction result from every decision tree.
(3) In this step, voting will be performed for every predicted result.
(4) At last, select the most voted prediction result as the final prediction result.

In calculation, R version 3.4.4 and randomForest function in randomForest package of R, which is a commonly used software for statistical analysis, were used for estimation and calculation. The number of feature values was determined from the results of simulation, changing the number of feature values, and the appropriate number was applied when PC was maximized. Mtry (number of feature values) was determined as 1. As for the number of trees, the default number of trees in randomForest, the R package, was used.

### 2.7. Modeling and Estimation of Estrus Behavior by Svm

An SVM, a supervised machine learning algorithm, could be used for both classification and regression challenges [42]. Support vector machine was applied to classification [43], pattern recognition [44] and so on. The objective of SVM algorithm is to find a hyperplane in an N-dimensional space that distinctly classifies the data points [45]. To separate the two classes of data points, there are many possible hyperplanes that could be chosen. Thus, it is needed to find a plance that has the maximum margin, i.e., the maximum distance between data points of both classes. Maximizing the margin distance provides some reinforcement so that future data points can be classified with more confidence [45]. Hyperplanes are decision boundaries that help classify the data points. Data points falling on either side of the hyperplane can be attributed to different classes. Also, the dimension of the hyperplane depends upon the number of features. Support vectors are data points that are closer to the hyperplane and influence the position and orientation of the hyperplane. Using these support vectors, the margin of the classifier is maximized. Deleting the support vectors will change the position of the hyperplane [45].

In the SVM algorithm, the margin between the data points and the hyperplane should be maximized. The loss function that helps maximize the margin is hinge loss:

$$c\left(x,y,f\left(x\right)\right)=\left\{\begin{array}{cc} 0 & \left(y*f(x)\geq 1\right)\\ 1-y*f(x) & \left(otherwise\right) \end{array}\right.$$

where *x* is observed value and *y* is estimated value. The cost is 0 if the predicted value and the actual value are of the same sign. If they are not, we then calculate the loss value. We also add a regularization parameter the cost function. The objective of the regularization parameter is to balance the margin maximization and loss. After adding the regularization parameter, the cost functions is defined as following:

$$\min_{w}\lambda {∥w∥}^{2}+\sum_{i=1}^{n}{\left(1-y_{i}\langle x_{i},w\rangle \right)}_{+}$$

Now that we have the loss function, we take partial derivatives with respect to the weights to find the gradients. Using the gradients, we can update our weights.

$$\frac{\delta }{\delta w_{k}}\lambda {∥w∥}^{2}=2\lambda w_{k}$$

$$\frac{\delta }{\delta w_{k}}{\left(1-y_{i}\langle x_{i},w\rangle \right)}_{+}=\left\{\begin{array}{cc} 0 & \left(y_{i}\langle x_{i},w\rangle \geq 1\right)\\ -y_{i}x_{ik} & \left(otherwise\right) \end{array}\right.$$

where λ is regularization parameter.

When there is no misclassification, the only thing to do is update the gradient from the regularization parameter.

w=w−α·(2λw)

when there is a misclassification, the loss along with the regularization parameter is included to perform gradient update.

$$w=w+\alpha \cdot (y_{i}\cdot x_{i}-2\lambda w)$$

In calculation, R version 3.4.4 and SVM function in the Kernlab package of R, which is a commonly used software for statistical analysis, were used for estimation and calculation. The number of feature values was determined from the results of simulation, changing the number of feature values, and the appropriate number was applied when PC was maximized. The radial basis function (RBF) kernel and cost parameters were determined from the results of a grid search, and the number of *k* for *k*-fold cross validation was determined based on Sturges’ formula:

k=1+log(n)/log2

where *n* is the sample size. In this paper, the sample size was 9600 (= 1200 × 8), and 14-fold cross validation was applied. Cross (cross validation), gammaRange (RBF kernel parameter) and costRange (cost parameter) was determined as 14, 10${}^{5}$ and 10${}^{2}$, respectively.

### 2.8. Modeling and Estimation of Estrus Behavior by Neural Network

Neural network which was a type of machine learning that modeled itself after the human brain, created an artificial neural network, that allowed the computer to learn, by incorporating new data via an algorithm [46]. Neural networks were applied to pattern classification [47], dynamic modeling and control [48], signal processing [49] and so on. In calculation, R version 3.4.4 and the nnet function in the nnet package of R, which is a commonly used software for statistical analysis, were used for estimation and calculation. The number of hidden layers was determined from the results of the simulation, changing the number of hidden layers, and the appropriate number was applied when PC is maximized. Size (number of hidden layers) is determined as 10.

### 2.9. Calculation of Percentage Concordance

In order to examine the reliability of machine learning estimation, their rate of concordance was estimated by focusing on the occurrence of estrus behavior (“approaching the male” and “standing near the male”) at 1200 time points and compared to that detected through human observation.

Concordance was an event during which the machine learning estimate of behaviors matched the occurrence of a social behavior, as determined by a human expert. Machine learning methods were allowed a two-point window for calculating the rate of concordance, where the event was considered to be concordant, if machine learning methods estimated a behavior to have occurred within the range of a time point (which corresponds to 0.5 s each), before or after the time point at which the behavior was noted by human observation. This was required to take into account the slight difference between human cognitive response and the corresponding machine response to a video image. A false negative (FN) was defined to be an event recorded as estrus behavior by the human observer but not by machine learning methods. Correspondingly, a false positive (FP) was an event recorded as estrus behavior by machine learning methods, and not by the human observer. The concordance at each of the 1200 time points was examined, and the percentage concordance (PC) was calculated using the following formula:

PC=TP/(TP+FN+FP)

where TP is the number of concordant events, FN is the number of false negative events, and FP is the number of false positive events.

## 3. Results and Considerations

**Figure 5:**
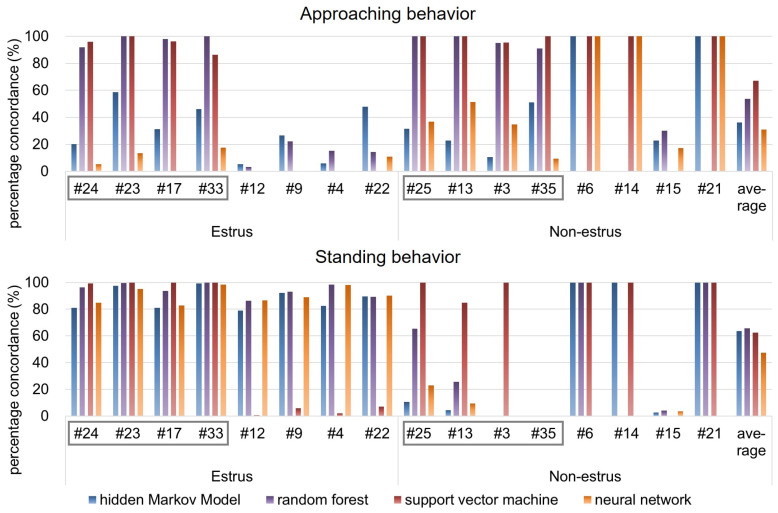
PC of each goat and average PC of all goats using various machine learning methods. Gray-colored frames represent goats used for training data.

The average PC between the results obtained from human observation and HMM analysis was calculated for 16 pairs of goats, including eight pairs of goats used as training data for the HMM. As for all machine learning methods, the PC of “approaching the male” state is shown in the upper of Figure 5, and that of “standing near the male” is shown in the bottom of Figure 5.

The concrete numerical values of PC, FN, and FP of HMM, random forest, SVM and NN are shown in Table 1, Table 2, Table 3 and Table 4, respectively.

At first, from Figure 5, it is found that the observations of approaching behavior were less than those of standing behavior. The following result and consideration are based on this.

As for “approaching the male” states estimated by HMM, from Figure 5 and Table 1, the minimum PC was 5.17%, the maximum PC was 58.54%, and the average PC was 30.16% for estrus goats. On the other hand, the minimum PC was 0.00%, the maximum PC was 100.00%, and the average PC was 42.36% for non-estrus goats. On the other hand, as for “standing near the male” state estimated by HMM, the minimum PC was 80.93%, the maximum PC was 99.30%, and the average PC was 87.74% for estrus goats. On the other hand, the minimum percentage is 0.00%, the maximum PC is 100.00%, and the average PC is 39.70% for non-estrus goats. Thus, it could be determined that the results of estimation by HMM reasonably emulated the results of estimation by human observation when “standing near the male” state of estrus goats would be detected.

As for “approaching the male” states estimated by random forest, from Figure 5 and Table 2, the minimum PC was 3.15%, the maximum PC was 100.00%, and the average PC was 55.59% for estrus goats. On the other hand, the minimum PC was 0.00%, the maximum PC was 100.00%, and the average PC was 52.02% for non-estrus goats. On the other hand, as for “standing near the male” state estimated by random forest, the minimum PC was 86.46%, the maximum PC was 100.00%, and the average PC was 94.54% for estrus goats. On the other hand, the minimum percentage is 0.00%, the maximum PC is 100.00%, and the average PC is 36.91% for non-estrus goats.

As for “approaching the male” states estimated by SVM, from Figure 5 and Table 3, the minimum PC was 0.00%, the maximum PC was 100.00%, and the average PC was 47.35% for estrus goats. On the other hand, the minimum PC was 0.00%, the maximum PC was 100.00%, and the average PC was 86.91% for non-estrus goats. On the other hand, as for “standing near the male” state estimated by SVM, the minimum PC was 0.58%, the maximum PC was 100.00%, and the average PC was 51.80% for estrus goats. On the other hand, the minimum percentage is 0.00%, the maximum PC is 100.00%, and the average PC is 73.13% for non-estrus goats.

As for “approaching the male” states estimated by neural network, from Figure 5 and Table 4, the minimum PC was 0.00%, the maximum PC was 17.65%, and the average PC was 5.90% for estrus goats. On the other hand, the minimum PC was 9.30%, the maximum PC was 100.00%, and the average PC was 56.21% for non-estrus goats. On the other hand, as for “standing near the male” state estimated by neural network, the minimum PC was 82.75%, the maximum PC was 98.44%, and the average PC was 90.63% for estrus goats. On the other hand, the minimum percentage is 0.00%, the maximum PC is 22.95%, and the average PC is 4.51% for non-estrus goats.

**Figure 6:**
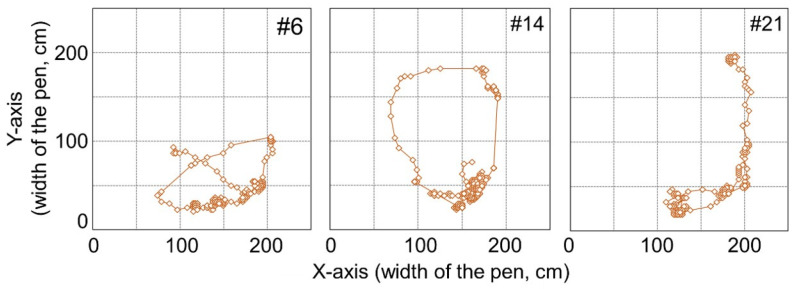
Tracking data of #6, #14 and #21 goats.

With regard to all machine learning methods, the combination of PC and FP for #6, #14, and #21 is either 100% and 0% or 0% and 100%. Trajectories of these goats are shown in Figure 6. From Figure 4 and Figure 6, it can be observed that trajectories of these goats are different from other non-estrus goats. These goats appear to stay around a narrow region of the paddock, whereas the other goats move around in a wide area. The human observer, through all the video frames, judges the behavior of these goats as a “behavior besides approaching and standing” state, not an “approaching the male” state or “standing near the male” state. If all frames were judged to be the “behavior besides approaching and standing” state using machine learning methods, the value would be 100%. However, the PC value was 0%, and FP was 100%, even if a few frames were judged as “approaching” or “standing.” These goats, #6, #14, and #21, are judged as shown above.

In total, the PC of the random forest and SVM are relatively high. However, we need to consider that #24, #23, #17, #33, #25, #13, #6, and #3 is a training data set. As for approaching behavior, the PCs of these goats are relatively high; however, the PCs of other goats are low. We think one reason is that the random forest and SVM tend to overfit the data, and the other reason is because the training dataset may not yet be sufficient to fit the variance that naturally occurs in the dataset with many individuals; thus, data from many more animals and with a wider variance in the signal associated to each behavior seem to be required for a good training dataset. Considering these reasons, the estimation of training data by random forest and SVM have good accuracy; however, the estimation of other data does not. Considering these reasons, the estimation of training data by random forest and SVM have good accuracy, however, the estimation of other data does not have accuracy. The estimation accuracy of the various goats would improve if the various goat’s behavior data were acquired. However, this is not realistic from the viewpoint of efficiency. The random forest and SVM are not an adequate machine learning method to estimate the goat’s behavior, considering this reasoning. This conclusion is a consideration of the “approaching behavior.” However, this also applies to “standing behavior” (this is discussed later). As for neural network, the PC value is low. The reason for this may be the lack of training data and machine learning not being efficient. Thus, this estimation is not a good result.

As for the “approaching behavior” of estrus goats, the PC of #12 and #4 are smaller than that of the other estrus goats. As shown in Figure 4, #12 and #4 stay in a narrow area near the male paddock, unlike other estrus goats. The trajectory beside the #4 goat is spread widely along the y-axis. However, #12 does not move in the x-axis direction much, which is different from the behavior of other goats, and #4 does not move in the y-axis direction much, which is different from the behavior of other goats. These are suggested that this causes the small PC value for #12 and #4.

As for the standing behavior of estrus goats, there is almost no difference between all the machine learning methods. However, PC of random forest and SVM are relatively high. According to actual data, the frame number of the standing behavior is much more than that of the approaching behavior, and, unlike the PC of #6, #14, and #21, misestimation does not affect the PC value much. Also, it is suggested that estrus goats tend to stay in a wide area near the male paddock; thus, they are easily detected.

On the other hand, for non-estrus goats, the PC is totally smaller than the approaching state and standing state of estrus, and the approaching state of non-estrus. The standing state of non-estrus goats rarely occurs, being present in a few frames out of 1800 frames. Thus, the PC decreases if misestimation occurs in a few frames out of 1800 frames. For non-estrus goats, the standing state occur far less than the approaching states. Besides, the training data are still few. Considering these, the PC is smaller than the approaching and standing state of estrus goats, and the approaching state of non-estrus goats. The PC of the random forest seems to be the highest among all machine learning methods; however, an overfitting problem exists, as stated above. HMM gets the large PC next to the random forest. HMM could model a Shiba goat’s behavior based on Markov probability and has the probability of characterizing Shiba goat’s behavior statistically, similar to that of mice [16,17].

From the above discussion, considering the PC, convenience of modeling, and computing time needed, we suggest that estimation by HMM is an adequate machine learning method for estimating the goat’s behavior. The PC of neural network is small. However, if additional data are acquired and learned by neural network, the PC of neural network would increase. If the amount of data is small, HMM is an adequate method for estimation; on the other hand, if the amount of data is large, neural network can be an adequate method for estimation.

As for SVM, we should also consider about computing time. It is said that SVM requires much time. Thus, it might not take a long time to estimate the goat’s state by SVM if the data are small, like those used in this study. However, it might take a long time to estimate if more data were used. This will be a problem if the goat’s state estimation system is considered for practical use.

**Figure 7:**
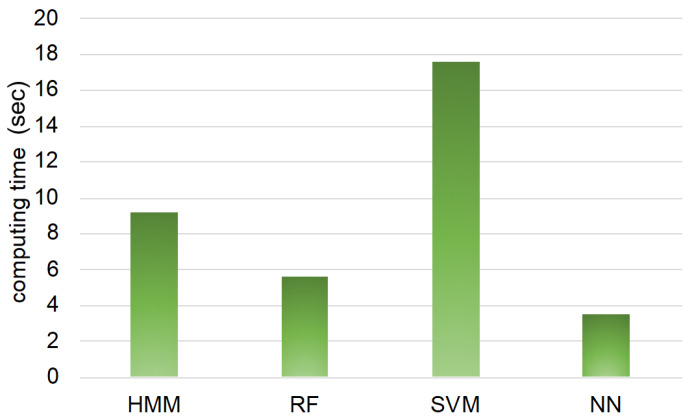
Computing time of each machine learning method.

As a reference, Figure 7 shows the computing time from modeling to estimation of all 16 goats by each machine learning method. From Figure 7, the neural network takes the shortest computing time, while SVM takes the longest. HMM takes a long computing time compared to SVM. This may be the difference in programing language: MATLAB is used for estimation by HMM, and R is used for estimation by random forest, SVM, and neural network. In this study, we use MATLAB for estimation by HMM because this was used in our previous study [10,16,17]; however, if HMM coded by R is used, the computing time of HMM may be shorter. Totally, the computing time of HMM, random forest, and neural network has little practical difference. Thus, SVM may be an inadequate method for estimating goats’ behaviors practically from the point of computing time, though SVM has a high PC value for estimating approaching and standing behaviors.

Table 5 shows the percentage of time spent in estrus and non-estrus behavior as determined by observation and all machine learning methods. From Table 5, as for estrus, from the result of the t-test, significant differences between observation and HMM in the determination of approaching behavior, and observation and SVM in the determination of standing behavior, were observed (*p* < 0.05). As for non-estrus, from the result of the t-test, significant differences between observation and neural network in the determination of approaching behavior, as well as observation and HMM and observation and neural network in the determination of standing behavior, were observed (*p* < 0.05). On the other hand, there was no significant difference between observation and RF. In other words, we could say that RF seems to resemble human observation well. Considering this totally, random forest seems to resemble human observation well and estimate goats’ behaviors similarly to a well-trained observer. However, as described above, there is the problem of overfitting or variance in the dataset. As for SVM, repeatedly, there is the problem of computing time. Totally, considering PCs, computing time, and the percentage of time spent in estrus and non-estrus behaviors, HMM may not be the best method, but it is adequate for estimating goat behavior at this point, though the PC of HMM is relatively low.

## 4. Conclusions

In this study, machine learning method as HMMs, random forests, SVMs, and neural networks, were applied to detect and estimate whether a goat, a typical mammal, was in estrus based on the goat’s behavior, and the adequacy of the method was verified. From calculations and analysis, the results of the estimation using HMM seemed to emulate the results of estimation by human observation reasonably well now. In particular, unlike other machine learning methods, the HMM has an advantage in that it is a time series model; thus, estimations by the HMM can clarify the detailed time series changes in a goat’s behavior and its underlying cause.

As for statistical modelling, the goat’s observable variable was considered as a Gaussian distribution. However, upon choosing to model the step length, we recommended using a standard parametric distribution, defined for positive numbers, such as the exponential, Weibull, or gamma distributions [50,51,52,53,54].

Poor PC values in some goats could have been caused by the choice of distribution modelling. Therefore, the application of these distributions as observable variables and verification of the validity of modelling are proposed. In addition, verification of interpretation of parameters biologically is also required and is proposed as a future study.

**Figure 8:**
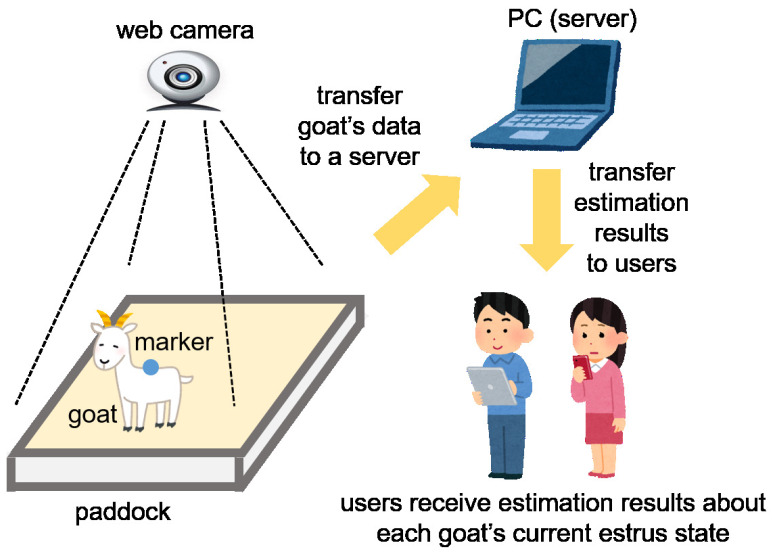
The proposed automated estrus estimation system for goats.

In the future, we aim to construct a system based on the results of this manuscript. Although this is only a concept at this stage, we would like to propose our system that uses IoT technology for estimating a goat’s estrus or non-estrus state using recorded behavior. Each goat is monitored from above by a camera. Data from the goat that can be detected by several methods are then transformed into numerical data. In this manuscript, each goat’s behavioral data is acquired by filming a marker attached to the goat’s back. This may be improved by acquiring data that was image-processed. The data is transferred to a server and the server then estimates the goat’s estrus state using the machine learning method. The estimation result gets sent to a user’s device, such as a tablet PC or smartphone, and the user can then see whether each goat is in estrus. We have constructed a similar system for estimating mice behavior [15], and our new proposed system is an application of this former system for estimating a goat’s behavior. The concept of our proposed system is shown in Figure 8. A trial system for goats has already been constructed [55]. We think that constructing this system can contribute to animal science, enabling the detection of animal states automatically and by increasing the efficiency of observers.

## Figures and Tables

**Table 1:** PCs, false positives, and false negative of estimation using HMM. Gray-colored cells represent goats used for training data.

		Approaching			Standing		
		PC	FP	FN	PC	FP	FN
	#24	20.18	77.13	2.69	80.93	9.76	9.31
	#23	58.54	21.95	19.51	97.40	2.26	0.34
	#17	31.13	49.06	19.81	81.13	17.61	1.26
Estrus	#33	46.00	52.00	2.00	99.30	0.00	0.70
	#12	5.17	94.83	0.00	79.03	0.00	20.97
	#9	26.44	66.83	6.73	92.16	3.70	4.14
	#4	5.98	92.39	1.63	82.47	0.38	17.15
	#22	47.87	13.83	38.30	89.51	10.49	0.00
	#25	31.62	18.97	49.41	10.56	76.40	13.04
	#13	22.87	11.85	65.29	4.32	93.69	1.99
	#3	10.55	13.91	75.54	0.00	100.00	0.00
Non-estrus	#35	50.98	33.33	15.69	0.00	100.00	0.00
	#6	100.00	0.00	0.00	100.00	0.00	0.00
	#14	0.00	0.00	100.00	100.00	0.00	0.00
	#15	22.87	37.22	39.91	2.73	96.82	0.45
	#21	100.00	0.00	0.00	100.00	0.00	0.00

**Table 2:** PCs, false positives, and false negative of estimation using random forest. Gray-colored cells represent goats used for training data.

		Approaching			Standing		
		PC	FP	FN	PC	FP	FN
	#24	91.84	0.00	8.16	96.21	3.79	0.00
	#23	100.00	0.00	0.00	99.54	0.46	0.00
	#17	98.04	0.00	1.96	93.56	6.44	0.00
Estrus	#33	100.00	0.00	0.00	100.00	0.00	0.00
	#12	3.15	94.49	2.36	86.46	0.19	13.35
	#9	22.22	40.74	37.04	93.05	6.84	0.11
	#4	15.15	63.64	21.21	98.39	1.61	0.00
	#22	14.36	59.41	26.24	89.14	7.67	3.19
	#25	100.00	0.00	0.00	65.45	34.55	0.00
	#13	100.00	0.00	0.00	25.76	74.24	0.00
	#3	95.07	1.10	3.84	0.00	100.00	0.00
Non-estrus	#35	90.91	0.00	9.09	0.00	100.00	0.00
	#6	0.00	100.00	0.00	100.00	0.00	0.00
	#14	0.00	100.00	0.00	0.00	100.00	0.00
	#15	30.15	19.40	50.45	4.07	95.93	0.00
	#21	0.00	100.00	0.00	0.00	100.00	0.00

**Table 3:** PCs, false positives, and false negative of estimation using support vector machines. Gray-colored cells represent goats used for training data.

		Approaching			Standing		
		PC	FP	FN	PC	FP	FN
	#24	96.08	3.92	0.00	99.37	0.63	0.00
	#23	100.00	0.00	0.00	99.88	0.12	0.00
	#17	96.36	3.64	0.00	99.81	0.19	0.00
Estrus	#33	86.36	13.64	0.00	100.00	0.00	0.00
	#12	0.00	30.00	70.00	0.58	0.00	99.42
	#9	0.00	0.00	100.00	5.80	0.34	93.86
	#4	0.00	0.00	100.00	2.03	0.00	97.97
	#22	0.00	6.90	93.10	6.96	0.00	93.04
	#25	100.00	0.00	0.00	100.00	0.00	0.00
	#13	100.00	0.00	0.00	85.00	15.00	0.00
	#3	95.32	0.83	3.86	100.00	0.00	0.00
Non-estrus	#35	100.00	0.00	0.00	0.00	100.00	0.00
	#6	100.00	0.00	0.00	100.00	0.00	0.00
	#14	100.00	0.00	0.00	100.00	0.00	0.00
	#15	0.00	0.00	100.00	0.00	0.00	100.00
	#21	100.00	0.00	0.00	100.00	0.00	0.00

**Table 4:** PCs, false positives, and false negative of estimation using neural networks. Gray-colored cells represent goats used for training data.

		Approaching			Standing		
		PC	FP	FN	PC	FP	FN
	#24	5.26	57.02	37.72	84.92	12.24	2.84
	#23	13.39	26.77	59.84	95.17	2.70	2.13
	#17	0.00	37.04	62.96	82.75	15.97	1.28
Estrus	#33	17.65	44.12	38.24	98.44	0.26	1.30
	#12	0.00	88.52	11.48	86.54	0.19	13.26
	#9	0.00	60.74	39.26	89.07	7.60	3.33
	#4	0.00	70.73	29.27	98.13	1.13	0.75
	#22	10.92	64.63	24.45	90.02	5.75	4.23
	#25	36.92	0.51	62.56	22.95	69.67	7.38
	#13	51.43	2.22	46.35	9.52	90.48	0.00
	#3	34.72	0.28	65.00	0.00	100.00	0.00
Non-estrus	#35	9.30	20.93	69.77	0.00	100.00	0.00
	#6	100.00	0.00	0.00	0.00	100.00	0.00
	#14	100.00	0.00	0.00	0.00	100.00	0.00
	#15	17.27	3.24	79.50	3.57	96.43	0.00
	#21	100.00	0.00	0.00	0.00	100.00	0.00

**Table 5:** Percentage of time spent in estrus and non-estrus behavior from observation and HMM. Gray-colored cells represent goats used for training data (unit: %).

		Approaching					Standing				
		Observation	HMM	RF	SVM	NN	Observation	HMM	RF	SVM	NN
	#24	4.08	2.33	2.42	3.83	5.50	65.17	74.67	68.00	65.33	69.42
	#23	7.75	4.08	6.33	7.25	3.83	71.5	73.42	71.92	71.5	70.42
	#17	4.25	0.25	2.67	4.5	2.50	43.58	53.67	46.25	43.42	50.08
Estrus	#33	1.58	1.08	1.25	1.83	1.33	95.75	95.75	95.67	95.17	93.67
	#12	0.58	6.08	10.08	0.25	4.50	85.83	71.42	63.17	0.08	70.17
	#9	5.33	4.08	4.00	0.00	8.25	73.00	76.83	73.25	1.58	69.17
	#4	1.00	8.17	1.83	0.00	2.42	65.75	58.75	65.08	0.50	63.92
	#22	6.75	9.67	10.67	0.50	12.92	71.83	73.50	69.58	2.17	69.00
	#25	16.00	8.08	14.08	15.92	4.83	3.00	5.17	4.58	3.00	8.33
	#13	25.67	16.25	22.67	25.42	8.92	1.33	14.33	5.42	1.67	18.92
	#3	0.00	0.00	0.08	0.00	0.00	0.00	0.00	0.00	0.00	0.17
Non-estrus	#35	29.83	26.83	28.67	28.83	10.00	0.00	7.75	0.67	0.00	21.00
	#6	0.00	0.92	0.17	0.00	0.00	0.00	0.00	0.25	0.00	0.08
	#14	22.33	16.25	7.50	0.00	1.75	0.50	9.33	14.08	0.00	16.08
	#15	0.00	0.00	0.17	0.00	0.00	0.00	0.00	0.00	0.00	0.00
	#21	2.75	3.42	1.75	2.17	0.83	0.00	5.08	2.08	0.33	4.75

## Abbreviations

The following abbreviations are used in this manuscript:

PC	percentage concordance
TP	true positive
FN	false negative
FP	false positive
HMM	hidden Markov model
RF	random forest
SVM	support vector machine
NN	neural network

## Appendix A. The Number of Observable Variables in This Model

The reason for adopting these three observable variables in this study was based on the results of a previous study [7]. Based on that study, the *x*-coordinate, *y*-coordinate, and step length of a goat were related to the goat being in estrus. However, it did not seem to be necessary to use all these parameters. Thus, some pairs of these observable variables were chosen, and the PC was calculated using a HMM, based on these same pairs of observable variables. In addition, the results of estimation between HMM and other machine learning methods were compared, and HMM seemed to be the most adequate of all estimation methods, from the point of modeling.
